# Evaluating the management trends for priapism and assessing the risk of priapism after in-office intracavernosal injections: a cross-sectional analysis

**DOI:** 10.1038/s41443-024-00861-2

**Published:** 2024-03-06

**Authors:** Joon Yau Leong, Zachary J. Prebay, David Ebbott, Michael Li, Paul H. Chung

**Affiliations:** 1 Department of Urology, Sidney Kimmel Medical College at Thomas Jefferson University, Philadelphia, PA USA; 2 Center for Digital Health and Data Science, Thomas Jefferson University, Philadelphia, PA USA

**Keywords:** Sexual dysfunction, Risk factors, Medical research

## Abstract

We describe the management trends of patients suffering from any priapism and evaluate the risks of developing priapism after intracavernosal injections (ICI) performed in office. We queried TriNetX for two separate male adult cohorts - those presenting with any priapism based on International Classification of Disease code, N48.3 (priapism) and those who underwent ICI in office based on Current Procedural Terminology code, 54235 (injection of corpora cavernosa with pharmacologic agent[s]). We evaluated treatment options for these patients after any priapism and described demographic risks for developing priapism after ICI performed in office. There were 17,545 priapism encounters and 26,104 usages of ICI in the office. Most common treatment for any priapism was corporal irrigation/injection of medications (11.3%). Patients presenting with priapism after ICI were younger (age > 65 years, OR 0.44 [95% CI 0.38–0.51], *p* < 0.01) and had a higher prevalence of mood disorders (20% vs 14%), behavioral disorders (7% vs 2%) and sickle cell disease (6% vs <1%). They were less likely to have diabetes (14% vs 22%), hypertension (33% vs 40%), prostate cancer (13% vs 25%) or have taken sildenafil or tadalafil (29–30% vs 35–38%). For patients administering ICI, proper screening and counseling of priapism is important to reduce complications.

## Introduction

Ischemic priapism is a urologic emergency that can lead to progressive corporal fibrosis and permanent erectile dysfunction (ED) if not treated in a timely fashion [1]. Some common etiologies for priapism include hematologic disorders such as sickle cell disease, illicit drug use, or pharmacologically-induced effects, particularly psychiatric medications [2–5].

One particular class of medication known to increase the risk of priapism are the components found in intracavernosal injections (ICI), such as papaverine, phentolamine, alprostadil, prostaglandin E1, or atropine [6, 7]. Due to this known but feared complication, appropriate dose titration of ICI medication may be performed with serial monitoring in the office prior to patients self-administering the medication at home [8]. Moreover, patients are counseled extensively regarding the signs and symptoms of priapism and the need to seek medical attention should this occur. Currently, there also exists guidelines published by the American Urological Association (AUA) to inform clinicians regarding the diagnosis and treatment options for acute ischemic priapism [9].

To date, research pertaining to current management trends for patients suffering from priapism are currently underway [10]. Herein, utilizing a global, research database, we aim to describe the overall management trends after any initial priapism encounters. We also evaluate the rates of priapism and patient demographics after ICI performed in the office.

## Materials and methods

### Patient population and index event

Our study was exempt from Institutional Review Board approval given the de-identified nature of the TriNetX registry. We queried the TriNetX Research network, containing 80 individual healthcare organizations at time of analysis, for two separate male adult cohorts ages 18 years and older. The first were those who presented with any priapism based on the International Classification of Diseases-10 (ICD-10) code, N48.3, which encompasses all priapism classifications including arterial priapism [11]. The second cohort were those who underwent ICI in the office, and these were identified using the Current Procedural Terminology (CPT) code, 54235. A full definition of each CPT and ICD-10 code utilized in our study is included in Supplementary Table 1. Analyses were performed on February 28th, 2023.

### Selected treatments after any index priapism encounters

We described the landscape of treatment options for patients with any priapism at the time of presentation, within 7 days week, within 90 days, within 1 year, and within 5 years after initial presentation in a cumulative fashion. We selected same day and 7 days to account for the acute treatment of priapism. We selected 90 days, 1 year, and 5 years after initial presentation to capture the delayed management of subsequent ED with penile implant placement. We descriptively identified treatments for priapism using commonly associated CPT codes (inflatable penile prosthesis 54405, malleable penile prosthesis 54400, irrigation and/or injection 54220 or 54235, saphenous vein shunt 54420, corporal shunt 54430, glans shunt 54435) and evaluated temporal trends for each individual management option.

### Risk of priapism after intracavernosal injections performed in the office

We performed descriptive statistics to assess rates of priapism after ICI within 3 days and between 4–90 days after any ICI injection done in the office for all causes. We selected 3 days to evaluate for potential priapism from an injection performed in the office and 4–90 days to evaluate for priapism secondary to early self-performed injections at home. We then compared demographic differences between patients presenting with priapism after ICI and those who did not. These demographic variables include age, race, ethnicity, body mass index (BMI), history of hypertension, diabetes mellitus, mental and behavioral disorders, mood disorders, childhood behavioral and emotional disorders, sickle cell disorders, sildenafil use, tadalafil use, and history of prostate cancer treated with radical prostatectomy (CPT 55840, 55845, 55866). Next, we then performed subgroup analyses for each individual risk factor (i.e., patients with diabetes vs patients without) and utilized all remaining variables for propensity score matching (PSM) at 30 days. A *p*-value of <0.05 was considered statistically significant.

## Results

### Selected treatments after any index priapism encounters

We identified a total of 17,545 recorded instances of any priapism events. Table 1 depicts the treatment options for patients presenting after priapism based on timeline in a cumulative fashion. The most common treatment modality, irrespective of timing of presentation, was corporal irrigation and/or injection of intracavernosal phenylephrine with 11.3% on the same day of priapism diagnosis presented and 12.5% within 7 days after priapism presented. While corporal aspiration is the most likely first step to be performed during a priapism encounter clinically (11.3%) and is by definition associated with CPT 54220, there was no separate CPT code associated with this procedure alone.

**Table 1:** Management trends of patients for priapism of any cause presented in a cumulative fashion.

Time of presentation	Corporal irrigation/injection (CPT 54220, 54235)	Corpora cavernosa-glans penis fistulization (CPT 54435)	Corpora cavernosa-corpus spongiosum shunt (CPT 54430)	Corpora cavernosa-saphenous vein shunt (CPT 54420)	Malleable penile prosthesis (CPT 54400)	Inflatable penile prosthesis (CPT 54405)
Same day	11.3%	0.66%	1.1%	0.30%	0.06%	0.11%
Within 7 days	12.5%	0.96%	1.9%	0.44%	0.33%	0.18%
Within 90 days	-	-	-	-	0.38%	0.51%
Within 1 year	-	-	-	-	0.43%	1.0%
Within 5 years	-	-	-	-	0.47%	1.4%

Other treatment options for any priapism include distal shunting (0.66% at presentation), proximal shunting (1.1% at presentation), and creation of corpora cavernosa to saphenous vein shunting (0.3% at presentation). Lastly, placement of malleable or inflatable penile prostheses were also options for the management of priapism. The percentage of penile prosthesis (PP) placement at time of priapism encounter was low at 0.06% for malleable implants and 0.11% for inflatable implants. Although this number did slowly increase over time after five years from presentation, it’s overall prevalence still remained low at 0.47% for malleable PP and 1.4% for inflatable PPs.

### Risk of priapism after intracavernosal injections performed in the office

We identified a total of 26,104 usages of ICI performed in the office, of which 4.0% patients did develop a priapism episode. The univariate analysis is highlighted in Table 2. We found that patients presenting with priapism after ICI in the office were younger (46.4 vs 57.4 years, *p* < 0.01), have lower BMIs (28.4 vs 29.0 kg/m^2^, *p* < 0.01), and had a higher prevalence of mood disorders (20% vs 14%, *p* < 0.01), behavioral disorders (7% vs 2%, *p* < 0.01) and sickle cell disease (6% vs <1%, *p* < 0.01). Conversely, these patients tend to have less comorbidities such as diabetes (14% vs 22%, *p* < 0.01), hypertension (33% vs 40%, *p* < 0.01) or a history of prostate cancer (13% vs 25%, *p* < 0.01). These patients were also less likely to have taken phosphodiesterase-5 inhibitors such as sildenafil (30% vs 35%, *p* < 0.01) or tadalafil (29% vs 38%, *p* < 0.01) in the past.

**Table 2:** Univariate analysis of risk factors for developing priapism within 3 days after intracavernosal injections in the office.

Variables	Priapism	No priapism	*p*-value
Age, year (mean)	46.4	57.4	<0.01
BMI, kg/m^2^ (mean)	28.4	29.0	<0.01
White	59%	58%	0.65
Not Hispanic or Latino	74%	71%	0.03
Hypertensive diseases (I10–I16)	33%	40%	<0.01
Diabetes Mellitus (E08–E13)	14%	22%	<0.01
Mental and behavioral disorders due to psychoactive substance use (F10–F19)	21%	13%	<0.01
Mood (affective) disorders (F30–F39)	20%	14%	<0.01
Behavioral and emotional disorders with onset usually occurring in childhood and adolescence (F90–F98)	7%	2%	<0.01
Malignant neoplasm of prostate (C61)	13%	25%	<0.01
Sickle cell disorders (D57)	6%	<1%	<0.01
Sildenafil use	30%	35%	<0.01
Tadalafil use	29%	38%	<0.01

Age and body mass index (BMI) were analyzed as continuous variables and evaluated using student’s *t*-test; all other remaining variables were analyzed as categorical variables and evaluated using Chi-squared test.

Of patients receiving ICI in the office who present with priapism, the majority of patients presented near-immediately, with 4% (*n* = 1027) occurring within the first 3 days after injection, and 1.6% (*n* = 415) occurring between days 4 and 90. The recurrence rates (2nd episode) for priapism was 32.0% and 35.1% within one and five years, respectively.

Table 3 highlights risk factors of priapism within 30 days after ICI performed in the office based on multivariate analyses after PSM. Although we found that a history of diabetes and being overweight (BMI > 25 kg/m^2^) were no longer associated risks for priapism, the rest of the clinical characteristics remained consistent with our hypotheses. Patients with a history of mental health disorders, mood disorders or childhood behavioral/emotional disorders were 3.1×, 1.8×, and 4.1× more likely to develop priapism after ICI; older patients (OR 0.44, 95%CI 0.38–0.51, *p* < 0.01), patients using phosphodiesterase-5 inhibitors (OR 0.75, 95%CI 0.66–0.85, *p* < 0.01), and those with a history of radical prostatectomy for prostate cancer (OR 0.61, 95%CI 0.47–0.79, *p* < 0.01) were all less likely to be at risk for priapism after ICI done in the office.

**Table 3:** Risk factors for priapism after intracavernosal injections (ICI) in the office based on multivariate logistic regression analyses after propensity score matching (PSM).

Risk factors	*N* after PSM		*N* with priapism	% priapism	OR (95% CI)	*p*-value
Hypertension	7096	No	274	3.9		
		Yes	358	5.0	1.32 (1.13–1.55)	<0.01
Diabetes mellitus	6273	No	288	4.6		
		Yes	329	5.2	1.15 (0.98–1.35)	0.09
BMI	5589	<25 kg/m^2^	354	6.3		
		>25 kg/m^2^	400	7.2	1.14 (0.98–1.32)	0.08
Phosphodiesterase-5 inhibitor use	10613	No	599	5.6		
		Yes	453	4.3	0.75 (0.66–0.85)	<0.01
Age	9644	<65 years	571	5.9		
		>65 years	258	2.7	0.44 (0.38–0.51)	<0.01
History of RP	3671	No	153	4.2		
		Yes	95	2.6	0.61 (0.47–0.79)	<0.01
Mental Health Diagnosis	4876	No	198	4.1		
		Yes	570	11.7	3.13 (2.65–3.70)	<0.01
Mood (affective) disorders	4756	No	259	5.4		
		Yes	448	9.4	1.81 (1.54–2.12)	<0.01
Childhood behavioral and emotional disorders	1034	No	59	5.7		
		Yes	206	19.9	4.11 (3.03–5.57)	<0.01

*BMI* body mass index, *RP* radical prostatectomy.

The TriNetX platform utilizes 1:1 greedy nearest-neighbor PSM, a statistical analysis technique that uses logistic regressions to generate balanced cohorts with similar effects of risk factors based on variable of interest. After PSM was performed, odds ratios (OR) were calculated, and confidence intervals (CI) were reported as 95% CI. A value of *p* < 0.05 was set as the cut-off for statistical significance.

## Discussion

### Treatment options and management trends for priapism of any cause

Current treatment options for any priapism range from procedures performed at the bedside to more invasive surgeries done in the operating room [9]. In our cohort, the most common initial treatment for priapism was corporal irrigation/injection (CPT 54220, 54235) at a rate of 11.3% when on presentation, which by definition also includes corporal aspiration. It may seem intuitive that this bedside procedure performed under local anesthesia is done most commonly and is consistent with current literature [12]. There is also a high likelihood that many of these cases resolve spontaneously without any need for procedural interventions. However, there may be a component of undercoding of corporal aspiration, which is a commonly performed initial step for all bedside priapism procedures, since it does not have a direct CPT code associated with it.

Subsequently, the most frequently coded procedures were proximal shunting (CPT 54430, 1.1%–2.3%) and distal shunting (CPT 54435, 0.66%–1.1%). This finding is interesting as the recent 2021 guidelines on acute ischemic priapism deems that there is inadequate evidence to quantify the benefit of proximal shunting in persistent priapism after distal shunts [9]. In prolonged cases, it is recommended that further studies such as penile Doppler ultrasound or repeat cavernosal-blood gas be obtained prior to considering proximal shunting [9]. Owing to the extent and rates of complications, including urethral strictures or urethrocutaneous fistulas, the consensus is that proximal shunts should only be considered after failure of more established, conservative measures [9]. Contemporary series suggests that rates for surgical management for any priapism ranges from 15%–17% [13–15]. The factors associated with surgical shunting of priapism depends on several clinical factors. Typically, patients requiring higher dosages of phenylephrine injected, those presenting with longer durations of prolonged erections, and those with a personal history of recurrent priapism [13, 14]. Lastly, an alternative surgical option should proximal or distal shunts fail is the cavernoso-saphenous shunt [16].

### Penile prosthetic implantation in the setting of priapism or associated erectile dysfunction

According to the AUA guidelines, clinicians can also consider placement of PP in select cases after discussing the risks and benefits of early versus delayed placement *(Expert Opinion)* [9]. Benefits of PP placement in refractory priapism include prevention of additional corporal fibrosis, maintenance of penile length, resolution of penile pain, and concurrent treatment of ED [17]. Currently, the prevalence of PP placement for refractory ischemic priapism are unknown. However, early intervention (within three weeks) is recommended compared to delayed placement as delaying insertion results in increased surgical complications (device erosion, malfunction, infection), and lower satisfaction rates with increased penile shortening [18–21]. Zacharakis et al. proposed the use of malleable PP for the preservation of penile length, ease of explanation should complications occur, and ability to exchange for an inflatable PP at a later time [19]. Conversely, Sedigh et al. proposed inflatable PP to avoid the need for these additional procedures and its associated costs and risks [20]. Temporal trends in our analysis indicate a slow increase in the number of prostheses being implanted over time, likely resulting from subsequent ED after priapism, with some PP being implanted as soon as the day of presentation. However, the overall prevalence remained low at 0.5–1.4%. Overall, recent literature demonstrates the role of PP implantation in select patients with ischemic priapism, to re-establish erectile function and decrease likelihood of penile shortening [17, 22].

While implantation of PP during the presentation of priapism is previously described, there are other clinical and logistical factors that need to be considered. Firstly, surgeon comfort level plays a major role in these situations. Priapism patients never present electively, and often present to hospitals that may not be a large, tertiary academic centers with fellowship-trained or high-volume prosthetic urologists. In such settings, the actual devices may not be readily stocked for emergent use. Next, delayed implantation after distal shunting may also allow the incisions to heal to reduce any further risk for device erosion and to decrease the need to undersize initial implants due to this concern. It also allows patients to have more time to consider and make an informed decision regarding undergoing malleable PP versus inflatable PP placement, even if it is within 2–3 weeks on discharge before significant corporal scarring and fibrosis sets in. Lastly, delaying PP placement also allows patients to undergo the necessary protocols for insurance authorization.

### Patient demographics and risk factors of priapism after intracavernosal injections in the office

ICI is commonly performed in the urology clinic for a variety of reasons. For example, evaluation of penile curvature during Peyronie’s disease, prior to performance of a penile Doppler ultrasonography, and ICI teaching for the treatment of ED. Previous reports comparing different ICI agents (prostaglandin E_1_, bimix, low-dose trimix, high-dose trimix) have demonstrated comparable efficacy, satisfaction, and complication rates among the different regimens utilized. A study by Bernie et al. did show that the rates of priapism were higher (23% vs 7.4%, *p* = 0.08), albeit not significantly, among patients receiving ICI via a risk-based approach rather than an empiric approach [8]. One of our findings demonstrate that 4% of patients develop priapism as a side effect of ICI performed in the office. Previous reports note an incidence of 2.7% of papaverine-induced priapism following penile color Doppler ultrasonography [6]. However, there is limited documentation in the literature with regards to timing and presentation of these patients, and these studies are typically done for ICI-induced priapism in the non-clinical setting [14, 15, 23, 24].

After the first episode of priapism, patients are also more likely to recur within the first year compared to the subsequent years up to five years. Although our analysis does not clearly delineate the setting by which the priapism recurrence occurs, it does demonstrate a high recurrence rate of 32% within the first year as opposed to an additional 3% for the subsequent four years (total 35.1%). This information can hopefully be useful whenever counseling patients regarding this disease, that patients with previous episodes of priapism are at risk for recurrence.

Our analyses found that demographics of patients presenting with priapism after ICI in the office tended to be younger with higher prevalence of psychiatric disorders. They likely have a stronger component of psychogenic ED with normal penile function and require lower or unpredictable doses of ICI medications to achieve a satisfactory erection. They may also have underlying psychosocial determinants such as mental health issues, including mood or pain disorders, that further increases this risk. This aligns with a study evaluating priapism encounters from a single-institutional, retrospective analysis by Zhao et al., where almost half the priapism encounters (49%) were attributed to recreational use of ICI without physician regulations [15]. This finding may potentially encourage providers to use penile duplex ultrasounds to objectively quantify flow or consider referring patients to a sexual therapist prior to initiating medical therapy with phosphodiesterase-5 inhibitors or ICI medications. Further efforts to reduce the risk of this harmful practice should be implemented by targeting this vulnerable population and by increasing public awareness. From a provider standpoint, these risk factors can help guide practitioners in the outpatient urology clinic during the administration of ICI for diagnostic or therapeutic use. Best practices should be implemented to reduce iatrogenic priapism events from ICI. This includes patient monitoring to ensure detumescence prior to discharge from clinic, counseling of precautionary signs and symptoms on discharge, and if necessary, use of ICI of phenylephrine to reverse any prolonged erections.

While the risk of sickle cell disease is a well-documented etiology for priapism, our analysis also found that patients with comorbidities including diabetes, hypertension, higher BMIs, and prostate cancer requiring radical prostatectomies have a decreased risk for developing priapism after ICI on the univariate analyses, while being diabetic or having a higher BMI was not predictive on multivariate analyses [25, 26]. Previous reports have demonstrated that younger patients with good erectile function and satisfactory clinical parameters on penile Doppler ultrasonography were the most likely to suffer from ischemic priapism after ICI [6, 23]. They hypothesize that younger men likely suffer from psychogenic ED, while risk factors such as diabetes and hypertension lead to vasculogenic pathologies as the main cause for ED. Also, men diagnosed with prostate cancer who subsequently undergo definitive therapy, such as radical prostatectomy, radiation or androgen deprivation therapy, suffer from ED secondary to iatrogenic causes. These etiologies are organic in nature and may in a way be “protective” of the risk of developing priapism. Patients with a previous history of phosphodiesterase-5 inhibitor usage were less likely to suffer from priapism with ICI as these patients may have undergone a stepwise approach to their ED management and have poorer erectile function, resulting in failed oral therapy and need for ICI. Nevertheless, concurrent use of phosphodiesterase-5 inhibitors and ICI is not uncommon, and previous studies have demonstrated that patients on tadalafil, rather than sildenafil, had a significantly higher rate of prolonged erection (>2 h), but not priapism (>4 h), especially during early titration phase [7]. This highlights the need for providers to ensure continued monitoring and education on injection techniques to decrease priapism risk especially when these two medication classes are used concurrently.

Our study is not without limitations. Similar to all registry studies, we had to rely on the data collection within this database, and assumptions were made regarding the accuracy of this large, de-identified dataset. There may also be a component of coding error or undercoding of certain ICD-10 or CPT codes. Next, we manually selected several risk factors, individual diagnoses, and procedural codes to evaluate and there is likely some inherent confounding since not all risk factors were not controlled for. Additionally, our analyses were limited to generic ICD/CPT codes, and we were unable to assess certain clinical characteristics, types of ICI molecules or dosages utilized, types of urology practices, and surgical approach, while PSM analyses was limited to 30 days. We were also not able to assess long-term follow-up outcomes of erectile function after priapism. For patients undergoing PP after priapism, we were unable to delineate between implantation purely due to priapism events vs priapism-induced ED. Also, we were only able to identify patients who received ICI in the office under provider supervision and these results may not necessarily be generalizable to those who self-perform ICI at home.

## Conclusions

This large, multi-institutional dataset enabled us to collect real-world data representing a range of practice settings and expertise. By leveraging a large global registry, we were able to evaluate the overall trends in management of any priapism encounters. We also describe the rates and demographics of patients with priapism after ICI performed in the office and identify risk factors with higher association for developing post-ICI priapism. For patients prescribed ICI, adequate counseling of the risks for priapism soon after injection is important to reduce damaging long-term consequences, such as ED, corporal fibrosis, and penile shortening.

## Supplementary information

Supplementary Table 1

## Data Availability

The datasets generated during and/or analyzed during the current study are publicly available within the TriNetX database.

## References

[1] Graham BA, Wael A, Jack C, Rohan MA, Wayne HJG. An overview of emergency pharmacotherapy for priapism. Expert Opin Pharmacother. 2022;23:1371–80. 35815373 10.1080/14656566.2022.2099271

[2] Schifano N, Capogrosso P, Boeri L, Fallara G, Cakir OO, Castiglione F, et al. Medications mostly associated with priapism events: assessment of the 2015–2020 Food and Drug Administration (FDA) pharmacovigilance database entries. Int J Impot Res. 2024;36:50–4. 35597798 10.1038/s41443-022-00583-3

[3] Gül M, Luca B, Dimitropoulos K, Capogrosso P, Milenkovic U, Cocci A, et al. What is the effectiveness of surgical and non-surgical therapies in the treatment of ischemic priapism in patients with sickle cell disease? A systematic review by the EAU Sexual and Reproductive Health Guidelines Panel. Int J Impot Res. 2024;36:20–35. 35941221 10.1038/s41443-022-00590-4

[4] Milenkovic U, Cocci A, Veeratterapillay R, Dimitropoulos K, Boeri L, Capogrosso P, et al. Surgical and minimally invasive treatment of ischaemic and non-ischaemic priapism: a systematic review by the EAU Sexual and Reproductive Health Guidelines panel. Int J Impot Res. 2024;36:36–49. 36151318 10.1038/s41443-022-00604-1

[5] Capogrosso P, Dimitropolous K, Russo GI, Tharakan T, Milenkovic U, Cocci A, et al. Conservative and medical treatments of non-sickle cell disease-related ischemic priapism: a systematic review by the EAU Sexual and Reproductive Health Panel. Int J Impot Res. 2024;36:6–19. 35995858 10.1038/s41443-022-00592-2

[6] Kilic M, Serefoglu EC, Ozdemir AT, Balbay MD. The actual incidence of papaverine-induced priapism in patients with erectile dysfunction following penile colour Doppler ultrasonography. Andrologia. 2010;42:1–4. 20078509 10.1111/j.1439-0272.2009.00940.x

[7] Furtado TP, Miranda EP, Deveci S, Jenkins L, Narus J, Nelson C, et al. Erectile response profiles of men using PDE5 inhibitors combined with intracavernosal injections as part of a penile rehabilitation program after radical prostatectomy. J Sex Med. 2023;21:29–32. 37973393 10.1093/jsxmed/qdad144 PMC12371532

[8] Bernie HL, Segal R, Le B, Burnett A, Bivalacqua TJ. An empirical vs risk-based approach algorithm to intracavernosal injection therapy: a prospective study. Sex Med. 2017;5:e31–6. 28190453 10.1016/j.esxm.2016.08.001 PMC5302379

[9] Bivalacqua TJ, Allen BK, Brock G, Broderick GA, Kohler TS, Mulhall JP, et al. Acute ischemic priapism: an AUA/SMSNA guideline. J Urol. 2021;206:1114–21. 34495686 10.1097/JU.0000000000002236

[10] Butaney M, Thirumavalavan N, Rodriguez D, Gross MS, Munarriz R. Current practice in the management of ischemic priapism: an anonymous survey of ISSM members. Int J Impot Res. 2019;31:404–9. 30718828 10.1038/s41443-019-0120-4 PMC6679808

[11] International Classification of Diseases, Tenth Revision, Clinical Modification (ICD-10-CM): Centers for Disease Control and Prevention; 2023. [updated June 29, 2023]. https://www.cdc.gov/nchs/icd/icd-10-cm.htm.

[12] Moussa M, Abou Chakra M, Papatsoris A, Dellis A, Peyromaure M, Barry Delongchamps N, et al. An update on the management algorithms of priapism during the last decade. Arch Ital Urol Androl. 2022;94:237–47. 35775354 10.4081/aiua.2022.2.237

[13] Palka J, DuComb W, Begun E, Soto-Aviles O. Factors associated with corporoglandular shunting for patients with first-time ischemic priapism. Urology. 2021;154:191–5. 33823171 10.1016/j.urology.2021.03.030

[14] Zhao H, Dallas K, Masterson J, Lo E, Houman J, Berdahl C, et al. Risk factors for surgical shunting in a large cohort with ischemic priapism. J Sex Med. 2020;17:2472–7. 33208295 10.1016/j.jsxm.2020.09.007 PMC8136145

[15] Zhao H, Berdahl C, Bresee C, Moradzadeh A, Houman J, Kim H, et al. Priapism from recreational intracavernosal injections in a high-risk metropolitan community. J Sex Med. 2019;16:1650–4. 31501058 10.1016/j.jsxm.2019.07.024 PMC7416696

[16] Mains E, Aboumarzouk O, Ahmad S, El-Mokadem I, Nabi G. A minimally invasive temporary cavernoso-saphenous shunt in the management of priapism after failed conservative treatment. Minim Invasive Ther Allied Technol. 2012;21:366–8. 22142185 10.3109/13645706.2011.636821

[17] Reddy AG, Alzweri LM, Gabrielson AT, Leinwand G, Hellstrom WJG. Role of penile prosthesis in priapism: a review. World J Mens Health. 2018;36:4–14. 29299902 10.5534/wjmh.17040 PMC5756805

[18] Tausch TJ, Zhao LC, Morey AF, Siegel JA, Belsante MJ, Seideman CA, et al. Malleable penile prosthesis is a cost-effective treatment for refractory ischemic priapism. J Sex Med. 2015;12:824–6. 25536880 10.1111/jsm.12803

[19] Zacharakis E, Garaffa G, Raheem AA, Christopher AN, Muneer A, Ralph DJ. Penile prosthesis insertion in patients with refractory ischaemic priapism: early vs delayed implantation. BJU Int. 2014;114:576–81. 25383397 10.1111/bju.12686

[20] Sedigh O, Rolle L, Negro CL, Ceruti C, Timpano M, Galletto E, et al. Early insertion of inflatable prosthesis for intractable ischemic priapism: our experience and review of the literature. Int J Impot Res. 2011;23:158–64. 21654814 10.1038/ijir.2011.23

[21] Yücel ÖB, Pazır Y, Kadıoğlu A. Penile prosthesis implantation in priapism. Sex Med Rev. 2018;6:310–8. 28916463 10.1016/j.sxmr.2017.08.002

[22] Moore J, Whelan TF, Langille GM. The use of penile prostheses in the management of priapism. Transl Androl Urol. 2017;6:S797–803. 29238659 10.21037/tau.2017.04.26 PMC5715174

[23] Güler Y, Erbin A. Independent predictive factors for occurrence of ischemic priapism after papaverine injection. Urol J. 2020;17:512–6. 32478401 10.22037/uj.v0i0.5890

[24] Pal DK, Biswal DK, Ghosh B. Outcome and erectile function following treatment of priapism: an institutional experience. Urol Ann. 2016;8:46–50. 26834401 10.4103/0974-7796.165717 PMC4719511

[25] Chrouser KL, Ajiboye OB, Oyetunji TA, Chang DC. Priapism in the United States: the changing role of sickle cell disease. Am J Surg. 2011;201:468–74. 21421100 10.1016/j.amjsurg.2010.03.017

[26] Idris IM, Burnett AL, DeBaun MR. Epidemiology and treatment of priapism in sickle cell disease. Hematol Am Soc Hematol Educ Program. 2022;2022:450–8. 10.1182/hematology.2022000380 PMC9820196 36485155

