# Supplementary material for: Evaluating the management trends for priapism and assessing the risk of priapism after in-office intracavernosal injections: a cross-sectional analysis
Source: Int J Impot Res. 2024 Mar 6;37(6):465–70. doi: 10.1038/s41443-024-00861-2 (PMC12185315; doi:10.1038/s41443-024-00861-2)
Supplement: Supplementary file 1 — Supplementary Table 1 [file 41443_2024_861_MOESM1_ESM.docx]

Supplementary Table 1: International Classification of Disease (ICD) and Current Procedural Terminology (CPT) codes and definitions

| Code | ICD or CPT | Definition |
| --- | --- | --- |
| I10-I16 | ICD | Hypertensive diseases |
| E08-E13 | ICD | Diabetes Mellitus |
| F10-F19 | ICD | Mental and behavioral disorders due to psychoactive substance use |
| F30-F39 | ICD | Mood (affective) disorders |
| F90-F98 | ICD | Behavioral and emotional disorders with onset usually occurring in childhood and adolescence |
| D57 | ICD | Sickle-cell disorders |
| 54220 | CPT | Irrigation of corpora cavernosa for priapism |
| 54235 | CPT | Injection of corpora cavernosa with pharmacologic agent(s) (eg, papaverine, phentolamine) |
| 54420 | CPT | Corpora cavernosa-saphenous vein shunt (priapism operation), unilateral or bilateral |
| 54430 | CPT | Corpora cavernosa-corpus spongiosum shunt (priapism operation), unilateral or bilateral |
| 54435 | CPT | Corpora cavernosa-glans penis fistulization (eg, biopsy needle, Winter procedure, rongeur, or punch) for priapism |
| 54400 | CPT | Insertion of penile prosthesis; non-inflatable (semi-rigid) |
| 54405 | CPT | Insertion of multi-component, inflatable penile prosthesis, including placement of pump, cylinders, and reservoir |
| 55840 | CPT | Prostatectomy, retropubic radical, with or without nerve sparing |
| 55845 | CPT | Prostatectomy, retropubic radical, with or without nerve sparing; with bilateral pelvic lymphadenectomy, including external iliac, hypogastric, and obturator nodes |
| 55866 | CPT | Laparoscopy, surgical prostatectomy, retropubic radical, including nerve sparing, includes robotic assistance, when performed |
